# Administration of *Alphitobius diaperinus* or *Tenebrio molitor* before meals transiently increases food intake through enterohormone regulation in female rats

**DOI:** 10.1002/jsfa.12305

**Published:** 2022-11-21

**Authors:** Alba Miguéns‐Gómez, Marta Sierra‐Cruz, Helena Segú, Raúl Beltrán‐Debón, Esther Rodríguez‐Gallego, Ximena Terra, Maria Teresa Blay, Anna Maria Pérez‐Vendrell, Montserrat Pinent, Anna Ardévol

**Affiliations:** ^1^ Departament de Bioquímica i Biotecnologia MoBioFood Research Group, Universitat Rovira i Virgili Tarragona Spain; ^2^ Monogastric Nutrition Centre Mas de Bover, IRTA Constantí Spain

**Keywords:** dietary protein, gut, enterohormones, food intake, insect

## Abstract

**BACKGROUND:**

It has been previously shown that acutely administered insect *Alphitobius diaperinus* protein increases food intake in rats and modifies the *ex vivo* enterohormone secretory profile differently than beef or almond proteins. In this study, we aimed to evaluate whether these effects could be maintained for a longer period and determine the underlying mechanisms.

**RESULTS:**

We administered two different insect species to rats for 26 days and measured food intake at different time points. Both insect species increased food intake in the first week, but the effect was later lost. Glucagon‐like peptide 1 (GLP‐1) and ghrelin were measured in plasma and *ex vivo*, and no chronic effects on their secretion or desensitization were found. Nevertheless, digested *A. diaperinus* acutely modified GLP‐1 and ghrelin secretion *ex vivo*.

**CONCLUSION:**

Our results suggest that increases in food intake could be explained by a local ghrelin reduction acting in the small intestine. © 2022 The Authors. *Journal of The Science of Food and Agriculture* published by John Wiley & Sons Ltd on behalf of Society of Chemical Industry.

## INTRODUCTION

Loss of appetite is a common symptom of several health conditions, and it is a frequent problem in the elderly,^1^ cancer patients^2^ (especially those undergoing chemotherapy),^3^ and also in children.^4^ The malnutrition caused by loss of appetite is an increasing cause of death in the population aged older than 75 years; however, it is a cause of health loss in the entire population. In all these cases the loss of appetite is always an aggravating factor for the diseases or situations that cause it, because reduced food intake leads to weight loss and the deterioration of general health.

Food intake is regulated through a series of complex signalling pathways involving central regulation and peripheral and intestinal signalling. In the intestine, nutrient and food components interact with the epithelium receptors and transporters to signal and regulate the feeding process. The enteroendocrine cells present in the gastrointestinal tract interact with nutrients and secrete hormones in response to food ingestion, acting as chemosensors of the lumen content.^5^ Enteroendocrine cells are scattered throughout the gastrointestinal tract, and the hormones they produce are concentrated in specific regions of the gut according to the role they play in regulating these physiological functions.^6^, ^7^ Ingested food triggers the release of different enterohormones. Cholecystokinin is a hormone secreted in the proximal small intestine that plays an important role in the digestion process that regulates gallbladder contraction and pancreatic enzyme secretion and also modulates intestinal motility and gastric emptying. It responds strongly to the presence of fat and protein in the lumen.^8^, ^9^ Glucagon‐like peptide 1 (GLP‐1) is an incretin hormone released in the distal small intestine and colon, and carbohydrates are a strong stimulus for its release. It is co‐localized in the colonic mucosa with peptide YY. The postprandial levels of these two hormones increase in plasma within 15–30 min in response to nutrients: carbohydrates and lipids are a strong stimulus for GLP‐1 secretion, and peptide YY is secreted in proportion to the caloric intake.^9^, ^10^ Moreover, ghrelin also modulates food intake. Its secretion rises in the preprandial state, promoting food intake, and falls rapidly after the ingestion of nutrients.^10^

We previously compared the effects of an acute administration of animal protein from insect (*Alphitobius diaperinus*) and beef and plant protein from almonds on the enterohormone secretion and food intake.^11^ We showed that insect, beef, and almond proteins modulate food intake differently when administered in a dosage with the same amount of protein 1 h before refeeding (beginning of the dark cycle). However, *A. diaperinus* is the only one of these proteins that increased food intake in rats. Using *ex vivo* pig and human intestinal samples we also showed that digested *A. diaperinus* modulated enterohormone release, resulting in a different secretome profile than beef and almond.

The search for new environmentally friendly produced protein sources is becoming more important. Therefore, according to our latest results working with insect protein, in this study we aimed to analyse whether the previously observed orexigenic effects of *A. diaperinus* were maintained for a longer period and whether they translated to body weight (BW) modulation. We also aimed to determine the mechanisms involved in these effects. In addition, we analysed whether the effect was species specific or whether it can be reproduced with other insect proteins.

## MATERIALS AND METHODS

### Chemicals and reagents

Buffalo worm (*A. diaperinus* larvae powder) was provided by Protifarm NV (Ermelo, Netherlands). *Tenebrio molitor* flour of subadult insects was obtained from Iberinsect, S.L. (Tarragona, Spain), and processed by FoodIE Research Group, University Rovira i Virgili, Spain. The nutritional composition of these samples is shown in Supporting Information Table S1.

Chemicals, porcine digestive enzymes (*α*‐amylase, pepsin, and pancreatin), bile salts, bovine serum albumin (fatty acid free), d‐glucose, d‐mannitol, amino acids, aprotinin, protease inhibitor cocktail (cOmplete™ ULTRA Tablets; Roche), and foetal bovine serum were purchased from Sigma‐Aldrich (Madrid, Spain). Amastatin was from Enzo Life Sciences (Madrid, Spain). Glutamine, penicillin, streptomycin, and Matrigel from Lonza (O Porriño, Spain).

The enzyme‐linked immunosorbent assay kits for total ghrelin (catalogue no. EZGRT‐91K) and total GLP‐1 (catalogue no. EZGLPT1‐36K) were purchased from Millipore (Billerica, MA, USA). Plasmatic parameters were measured with commercial kits according to manufacturer's instructions: insulin with an insulin enzyme‐linked immunosorbent assay kit (catalogue no. EZRMI‐13K) from Millipore (Madrid, Spain), and glucose triglycerides and cholesterol from QCA (Amposta, Spain). The Pierce BCA Protein Assay kit was from ThermoFisher (Waltham, MA, USA).

### Animals

We used 24 female RccHan:WIST rats (8 weeks old, 220–240 g) purchased from Janvier (Castellar del Vallés, Spain) and six male Wistar rats (used for the *ex vivo* experiment with duodenum). Most of these animals were housed at the animal housing facility of the University Rovira i Virgili. All rats were housed under standard conditions. For the RccHan:WIST rats, the animals were housed upon arrival in pairs for a week and then individually in animal quarters for another week to get them used to the voluntary oral administration. The rats had free access to food (standard chow, Teklad2014 from ENVIGO), containing (by energy) 20% protein, 13% fat, and 67% carbohydrates, and tap water. The room temperature was kept at 22 °C with a 12 h light/12 h dark cycle (lights from 7:00 a.m. to 7:00 p.m.). The animals were used in the experiments after this 2‐week acclimation period. All procedures were approved by The Animal Ethics Committee of University Rovira i Virgili (Tarragona, Spain) (CEA‐OH/10715/3).

### Experimental protocol for animal treatments

Animals were divided into three groups (*n* = 8 each). We tested two different insect protein sources, *A. diaperinus* and *T. molitor*, at a dose of 300 mg of protein per kilogram BW dissolved in water. The treatment consisted uniquely of raw insect flour mixed with tap water. The control group was administered an equivalent volume of tap water. To perform the treatments, animals were food deprived starting 3 h before the treatment, and at 6:00 p.m. animals received the insect protein load or water (control) by controlled voluntary oral intake with a syringe. At 7:00 p.m., when the dark cycle began, they had free access to food again. Treatments started at day 0 and were performed daily for 26 days, with two short breaks in between, of 2 days each, at days 12 and 20. In this experiment we aimed to analyse the effects of the incorporation of insect protein into the diet in a more realistic way. That is why we decided to include two breaks in the insect administration, since people can change their dietary routines eventually. Food intake was measured daily at 20 h after the dark cycle began. BW was measured once a week. At day 13, a plasma sample 20 min after the daily insect dose was obtained from the saphenous vein and immediately frozen for further analysis of the biochemical parameters. At the end of the study the animals were fasted for 12 h and euthanized by exsanguination (pentobarbital dose of 100 mg kg^−1^ BW for previous sedation). The blood was collected using tubes covered with ethylenediaminetetraacetic acid (Deltalab, Barcelona, Spain) as anticoagulant. Plasma was obtained by centrifugation (4500 × *g*, 15 min, 4 °C) and stored at −80 °C until analysis. A 0.5 mL blood aliquot to measure ghrelin was collected in a separate tube containing hydrochloric acid (0.1 mol L^−1^ in the final concentration) and protease inhibitors and then centrifuged to obtain the plasma (15 000 × *g*, 15 min, 4 °C). The intestine was carefully excised, washed with cold phosphate‐buffered saline (PBS) buffer and the different segments were measured and identified. Pieces of duodenum and distal jejunum were cut and used for *ex vivo* experiments. The rest of the tissue was cut into segments, immediately placed in liquid nitrogen, and then stored at −80 °C for further analysis. The different white adipose tissue depots were weighed. Supporting Information Fig. S1 shows the experimental design and the analysis performed.

### *Ex vivo* experiments with intestinal segments

Two *ex vivo* experiments were performed: (1) a study of the distal jejunum secretory responses obtained from animals treated with *A. diaperinus* and from control animals, as mentioned earlier herein; (2) another experiment with duodenum from another set of rats (mentioned earlier) to analyse the secretory response of this segment to acute *A. diaperinus* stimulation.

1. After washing the intestinal tube, the outer muscular layer of the distal jejunum segments was removed from the serosa layer with a scalpel. The tube was then sliced longitudinally and circles of tissue with a diameter of 5 mm were taken using a biopsy punch. The sample was kept at a low temperature with cold PBS buffer and an ice‐cold bath during the entire procedure. We then started the secretion study. We placed each distal jejunum circle in a well (48‐well plate) containing 0.4 mL of Krebs–Ringer bicarbonate (KRB) buffer with 10 mmol L^−1^ d‐mannitol pre‐warmed to 37 °C for 15 min. After this pre‐incubation period, the buffer was replaced by KRB buffer with 10 mmol L^−1^ glucose to study baseline secretion, or by pre‐warmed intestinally digested *A. diaperinus* dissolved in KRB buffer containing 10 mmol L^−1^ glucose to study the response to an acute insect stimulation. Samples were digested as previously described^11^ according to the INFOGEST harmonized protocol^12^ first published in 2014 by Minekus *et al*.^13^ The protein content of the digested sample was adjusted to a dose of 10 mg mL^−1^. The incubation period was 30 min. All the buffers used to incubate the tissue were previously oxygenated (95% oxygen, 5% carbon dioxide) for at least 15 min. Two replicates of each treatment were performed for each rat. All the treatments contained a cocktail of protease inhibitors, 100 kIU aprotinin, 10 μmol L^−1^ amastatin, and 0.1% fatty acid free bovine serum albumin. After the incubation period, the medium was collected in different aliquots and stored at −80 °C for enterohormone quantification.
2. After a short fasting period (1–3 h), the rats were euthanized by exsanguination (pentobarbital dose of 100 mg kg^−1^ BW for previous sedation) and their intestines were excised. Duodenum segments were collected and the experiment performed as in (1), except that the insect treatment was performed this time with 10 mg protein per millilitre of gastric‐digested *A. diaperinus*.

### GLP‐1 secretion test in GLUTag cells

The GLUTag cells used in this study were kindly donated by Professor Staels (University Lille, Institut Pasteur de Lille, Lille, France) with permission from Professor Drucker (Lunenfeld‐Tanenbaum Research Institute, Toronto, ON, Canada). The medium where the cells were cultured was composed by Dulbecco's modified Eagle's medium containing 1 g L^−1^ d‐glucose, supplemented with 10% foetal bovine serum, 100 U mL^−1^ penicillin, 100 mg L^−1^ streptomycin, and 1% of glutamine (final concentration 2 mmol L^−1^). The cells were incubated under a 5% CO2‐humidified atmosphere at 37 °C.

For the treatments, GLUTag cells were plated onto 24‐well plates precoated with Matrigel at a cell density of 200 000 mL^−1^ 24 h before the secretion study. Cells were then washed twice with PBS buffer and treated for 2 h with *A. diaperinus*, a mix of amino acids, or vehicle. To test the effect of *A. diaperinus* on GLP‐1 secretion, the intestinally digested *A. diaperinus* sample was dissolved in 4‐(2‐hydroxyethyl)‐1‐piperazineethanesulfonic acid (HEPES) buffer (1.25 mmol L^−1^) and tested at a protein concentration of 5 mg mL^−1^. To test the effects of the most abundant amino acids found in the intestinally digested *A. diaperinus*,^14^ we prepared a solution in HEPES buffer containing alanine, phenylalanine, proline, tyrosine, valine, and serine at a concentration equivalent to that found in 5 mg mL^−1^ of the intestinally digested *A. diaperinus* (0.36 mg mL^−1^, 0.31 mg mL^−1^, 0.35 mg mL^−1^, 0.46 mg mL^−1^, 0.28 mg mL^−1^, and 0.29 mg mL^−1^ respectively). The vehicle (HEPES buffer) was used to measure baseline secretion. All the treatments were performed in duplicate in each cell plate and repeated for three passages. After the treatment, the medium of each well was collected and stored at −80 °C in aliquots of 200 μL until the determination of total GLP‐1. Next, the cells were lysed with radioimmunoprecipitation assay buffer, and lysates were stored at −80 °C. They were then used to analyse total protein content with a BCA kit.

### Quantitative real‐time reverse transcription polymerase chain reaction analysis

Total RNA and complementary DNA were obtained as previously defined.^15^ Quantitative polymerase chain reaction amplification was performed using specific TaqMan probes from Applied Biosystems (Waltham, MA, USA): Rn00562406_m1 for the GLP‐1 receptor gene (*Glp1r*), and Rn00821417_m1 for the ghrelin receptor gene, also known as Growth Hormone Secretagogue Receptor (*Ghsr*). The relative expression of each gene was compared with the control group using the $2^{-\Delta \Delta C_{t}}$ method, with peptidylprolyl isomerase A gene (*Ppia*) expression (Rn00690933_m1) as a reference.

### Statistical analysis

The results are expressed as the mean ± the standard error of the mean. The sample size *n* for each variable is indicated in the corresponding figure description. Student's *t*‐test was used to compare the treatments with the control or vehicle group. One‐way analysis of variance followed by Bonferroni's *post hoc* test was used for multiple comparisons when comparing more than two groups. Normality of the data was evaluated with the Shapiro–Wilk test. *P*‐values <0.05 were considered statistically significant. These calculations were performed using XLSTAT 2021.2.1 software (Addinsoft, New York, NY, USA).

## RESULTS

### Food‐intake‐promoting effects of *A. diaperinus* are limited in time

**Figure 1:**
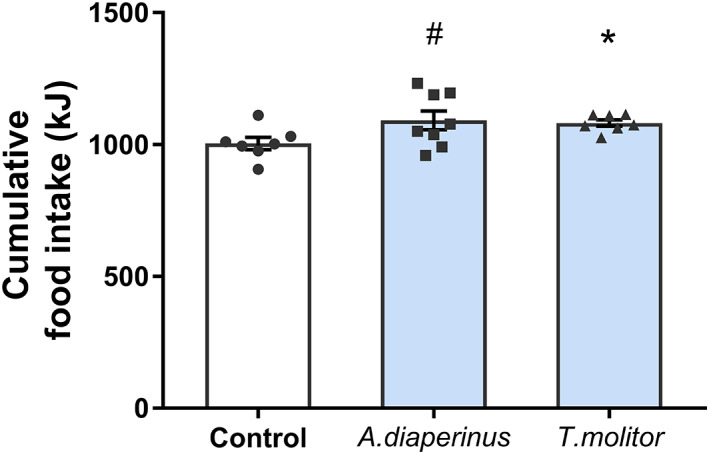
The 5‐day cumulative food intake within the first week after daily oral administration of an *Alphitobius diaperinus* or *Tenebrio molitor* dose (300 mg protein per kilogram body weight). The 24 h food intake was measured during the experiment after the beginning of the dark cycle. The control dose was an equivalent volume of tap water. The sample size was *n* = 8 rats per group. The results are expressed as the mean ± standard error of the mean. *, *P* < 0.05; #, *P* < 0.1, t‐test.

To study the effects of a long administration of *A. diaperinus* in rats, we administered protein at 300 mg kg^−1^ BW daily, with two short breaks in between (2 days). To test whether the previously shown acute effects of *A. diaperinus* on increasing food intake were maintained after a more prolonged administration, food intake was measured at different time points during the experiment. Figure 1 shows that the administration of both insect proteins increased the 5‐day cumulative food intake during the first week of treatment. However, the most effective one was the *T. molitor*, since the administration of the insect *A. diaperinus* only showed a tendency towards an increase in food intake during the first week (*P* = 0.012 and *P* = 0.070 *versus* the control group for the *T. molitor* and the *A. diaperinus* groups respectively). These effects were lost afterwards, since 3‐day cumulative food intake in the second week did not show significant differences for any of the insect‐administered groups (603.39 ± 20.61 kJ, 629.53 ± 21.97 kJ, and 641.36 ± 33.01 kJ for the control, *A. diaperinus*, and *T. molitor* groups respectively). These results suggest that the reported effects were not species specific.

This increased food intake was accompanied by a significantly higher percentage of BW gain during the first week in the case of *T. molitor* (*P* = 0.008), but not for *A. diaperinus* (*P* = 0.5) (1.834 ± 0.39%, 2.693 ± 1.16%, and 3.827 ± 0.51% of BW gain in the control, *A. diaperinus*, and *T. molitor* respectively). The feed efficiency ratio at this time point was also significantly increased in *T. molitor* but not in *A. diaperinus* (0.034 ± 0.005 g BW, 0.059 ± 0.007 g BW, 0.040 ± 0.017 g BW increase per kilocalorie in control, *A. diaperinus*, and *T. molitor* respectively). Moreover, in the *T. molitor* group, BW gain was still significantly increased at week two, and a tendency remained at the end of the experiment (Table 1). However, at sacrifice, the visceral adiposity of the control and insect‐fed animals was not significantly different. Fasting biochemical parameters on the sacrifice day (glucose, triglycerides, and cholesterol) showed no differences among the different groups (Table 1).

**Table 1:** Parameters at sacrifice

Parameter	Control	*Alphitobius diaperinus*	*Tenebrio molitor*
Body weight gain (%)	9.42 ± 1.0	9.80 ± 1.0	13.98 ± 2.8*
Visceral adiposity^a^ (%)	4.38 ± 0.14	4.26 ± 0.25	5.12 ± 0.64
Glucose (mmol L^−1^)	7.17 ± 0.15	7.34 ± 0.14	7.21 ± 0.38
Triglycerides (mmol L^−1^)	0.18 ± 0.03	0.17 ± 0.01	0.22 ± 0.05
Cholesterol (mmol L^−1^)	0.44 ± 0.01	0.48 ± 0.03	0.46 ± 0.03

*Note*: Values represent mean ± standard error of the mean of eight animals per group.

* *P* ≤ 0.1 *versus* control rats.

^a^ Visceral adiposity represents the weight percentage of mesenteric, retroperitoneal, and periovarian adipose tissues with respect to the total body weight.

### *A. diaperinus* treatment increases GLP‐1 secretion acutely but does not alter enteroendocrine signalling chronically

**Figure 2:**
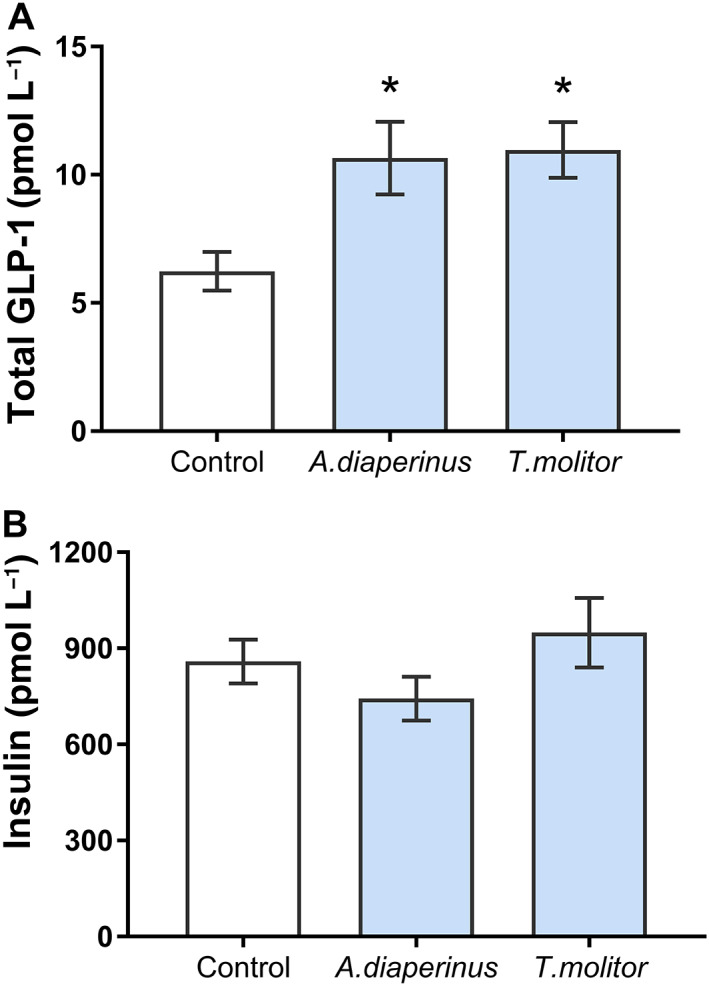
(A) Total glucagon‐like peptide 1 (GLP‐1) and (B) insulin were measured in plasma collected from saphena after 20 min of the *Alphitobius diaperinus* or *Tenebrio molitor* (300 mg protein per kilogram body weight) load. These samples were obtained at day 13 of the experiment. The control was an equivalent volume load of tap water. The sample size was *n* = 8 rats per group. The results are expressed as the mean ± standard error of the mean. *, *P* < 0.05, *t*‐test.

We tested the *in vivo* effects of an *A. diaperinus* treatment on the enterohormone signalling system. The effect of both insect loads on total plasmatic GLP‐1 was assayed at day 13. Figure 2(A) shows that 20 min after the insect load the total GLP‐1 significantly increased compared with the water‐loaded controls. At this time point, the insulin was not modified in these animals (Fig. 2(B)), and nor was glucose (3.86 ± 0.03 mmol L^−1^, 3.85 ± 0.07 mmol L^−1^, and 3.82 ± 0.05 mmol L^−1^ for the control, *A. diaperinus*, and *T. molitor* groups respectively).

**Figure 3:**
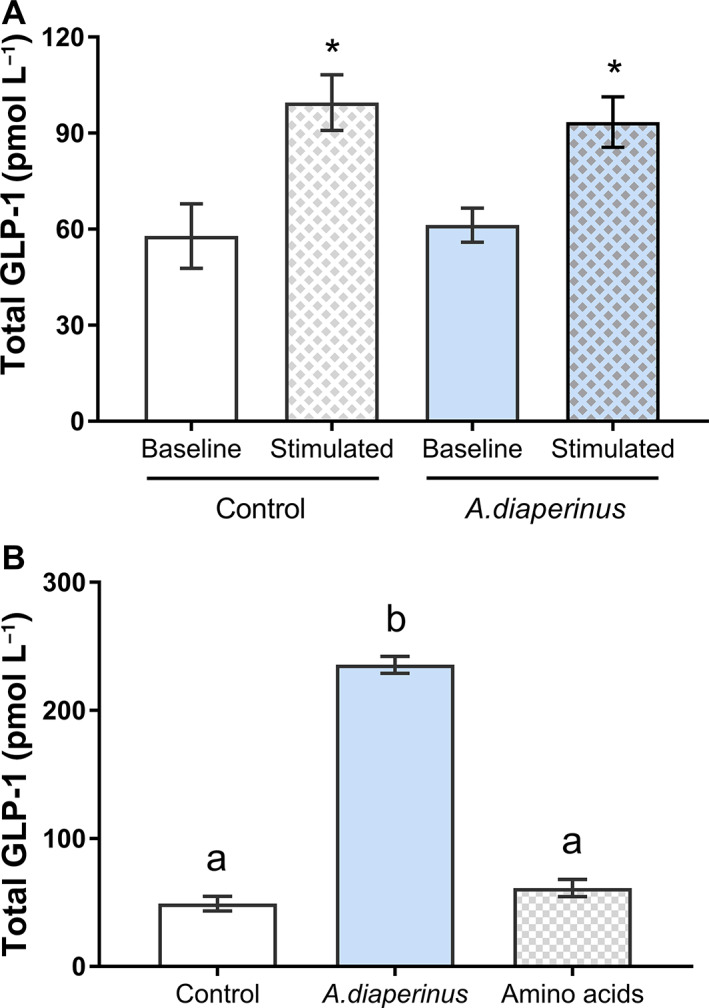
Total glucagon‐like peptide 1 (GLP‐1) secreted to the medium: (A) from distal jejunum explants obtained at sacrifice from control and *Alphitobius diaperinus* rats in baseline condition (vehicle treated) or stimulated condition (treated with 10 mg protein per millilitre of intestinally digested *A. diaperinus* for 30 min), *n* = 8 rats each condition; (B) from GLUTag cells, treated with 5 mg protein per millilitre of *A. diaperinus* or amino acid mixture for 2 h, *n* = 6, from three different passages. Results are expressed as the mean plus/minus standard error of the mean. *, *P* < 0.05 *versus* control, *t*‐test. Different letters (a, b) indicate significant differences (*P* < 0.05).

The effects of the chronic treatment with *A. diaperinus* were analysed using distal jejunum explants. On the day of the sacrifice, explants were obtained to measure baseline secretion and the acute response to intestinally digested *A. diaperinus*. Figure 3(A) shows that baseline total GLP‐1 secretion did not change between the control and insect‐fed animals. However, the acute stimulation of the explants with intestinally digested insect significantly increased the total GLP‐1 secretion to the medium both in the control and in the rats fed *A. diaperinus* for 26 days. Then, we checked any effect on reception system of GLP‐1. The secretion results are in accordance with the fact that we did not find any modifications of the messenger RNA levels from the GLP‐1 receptor locally in the ileum (1.02 ± 0.1 and 1.49 ± 0.5 for control and *A. diaperinus* respectively) or in the central system by measuring the expression levels in the hypothalamus (1.11 ± 0.21 and 1.42 ± 0.18 for control and *A. diaperinus* respectively). This suggests that there were no desensitization effects due to the chronic treatment.

We worked with GLUTag cells to test whether the acute GLP‐1 stimulation was due to direct effects on enteroendocrine cells. Figure 3(B) shows that digested *A. diaperinus* at a protein concentration of 5 mg mL^−1^ significantly increases total GLP‐1 release. We then tried to decipher whether the observed effects were mainly caused by the protein. To do so, we compared the effects of the digested sample with that of a mixture of the most abundant amino acids found in this digested sample,^14^ maintaining their concentrations (as described in the Materials and Methods section). Figure 3(B) shows that this mixture of amino acids alone was not responsible for the GLP‐1 secretory effect observed for *A. diaperinus*.

### *A. diaperinus* reduces acute ghrelin secretion in rat jejunum, showing no chronic effects on ghrelin release

Next, the effects on ghrelin secretion were tested. Ghrelin levels were analysed at day 13, 20 min after the daily insect load. There were no differences between the control and *A. diaperinus* groups (Table 2). At sacrifice, under fasting conditions, ghrelin levels remained the same in both groups.

**Table 2:** Total ghrelin plasmatic levels and messenger RNA (mRNA) levels of the ghrelin receptor, in the ileum and hypothalamus, for control and *Alphitobius diaperinus* rats

	Control	*A. diaperinus*
Total ghrelin (ng mL^−1^)		
Day 13	2.36 ± 0.1	2.63 ± 0.2
Day of sacrifice	1.81 ± 0.1	1.74 ± 0.1
mRNA level for Ghrelin‐R		
Hypothalamus	1.08 ± 0.19	1.13 ± 0.13
Ileum	1.07 ± 0.2	2.94 ± 0.9*

*Note*: On day 13, blood samples were collected 20 min after the treatments. Results are expressed as the mean ± standard error of the mean. Student's *t*‐test.

* *P* ≤ 0.1 *versus* control rats.

**Figure 4:**
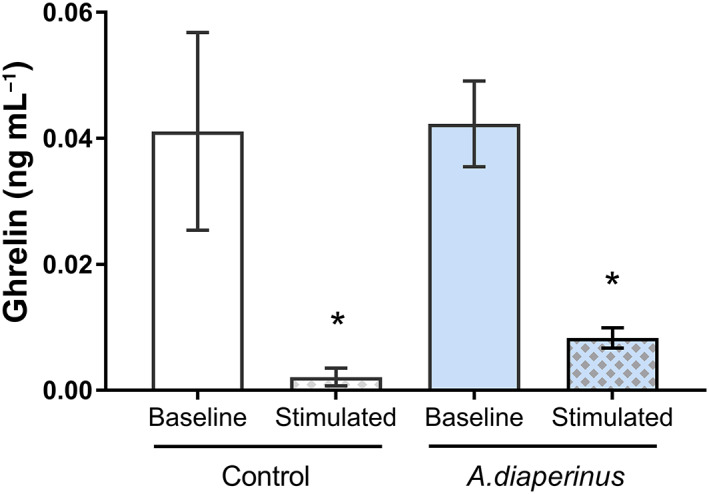
Total ghrelin secreted to the medium from distal jejunum explants obtained at sacrifice from control and *Alphitobius diaperinus* rats in baseline condition (vehicle treated) or stimulated condition (treated with 10 mg protein millilitre of intestinally digested *A. diaperinus* for 30 min); *n* = 8 rats for each condition. Results are expressed as the mean plus/minus standard error of the mean. *, *P* < 0.05 *versus* baseline (*t*‐test).

Total ghrelin release was measured in the media of the distal jejunum explants obtained at sacrifice, both under baseline conditions and after stimulation with digested *A. diaperinus*. Similar to what was observed for GLP‐1, we found no difference in baseline ghrelin secretion between the control and the rats treated with the insects for 26 days. However, explant stimulation with digested *A. diaperinus* led to a significant reduction in ghrelin release in both rat groups (Fig. 4). To understand the sensitivity to ghrelin, we analysed ghrelin receptor expression in the hypothalamus and ileum to check any central or local effects. Table 2 shows that though there were no central effects of *A. diaperinus*, the ileal ghrelin receptor tended to be increased in rats administered *A. diaperinus* compared with the controls.

Given the low levels of ghrelin obtained after treating the distal jejunum with the digested *A. diaperinus*, we aimed to reproduce this acute secretion study in an intestine fragment where ghrelin is more abundantly expressed; that is, the duodenum.^16^ We obtained *ex vivo* duodenal samples from six different rats and treated them with 10 mg protein per millilitre of gastric‐digested *A. diaperinus* for 90 min. Surprisingly, in this case we did not find a reduction of ghrelin secretion (1.31 ± 0.3  ng mL^−1^ and 2.87 ± 0.8 ng mL^−1^ in the vehicle and *A. diaperinus*‐treated explants respectively; *P* = 0.12, *t*‐test).

## DISCUSSION

We previously compared the effects of an equivalent protein load of beef, insect (*A. diaperinus*), and almond and found that they exerted a different enterohormone secretome, which led to differences in food intake.^11^ Our results showed that, surprisingly, only *A. diaperinus*, which reduced ghrelin secretion and increased GLP‐1 in pig and human intestinal segments, was able to stimulate energy intake when administered to rats as an acute load 1 h before refeeding. In the present study we aimed to analyse whether these effects could be extended for several days and determine the mechanisms behind them.

Here, we show that the eating‐promoting effects of the 300 mg kg^−1^ BW of an *A. diaperinus* protein load are observed during the initial days of the treatment, but these effects are lost later on. Our data on weekly BW gain and the food efficiency ratio during the first week were not statistically different from the control group. In a small human study, in subjects under resistance training, supplementation with a bar containing *A. diaperinus* protein led to a trend towards a reduced total energy intake compared with supplementation with an isoenergetic carbohydrate‐rich bar; however, these results are based on a short (3 days) weighed‐dietary record period, and the administration protocol did not establish any relationship with food intake.^17^ *T. molitor*, from the same family as *A. diaperinus* (Tenebrionidae), is a more studied insect, and was therefore also included in the present study. Our results show that it behaved very similarly to *A. diaperinus* in terms of promoting food intake, but it also resulted in significantly increased BW gain and feed efficiency. In a similar experiment, the BW of rats treated with different doses of *T. molitor* for 28 days was not modified by the 300 mg kg^−1^ BW dose but showed a tendency to increase with the 1000 and 3000 mg kg^−1^ BW doses. This seemed to be related to the food consumption in males, but not females.^18^ In Wistar Kyoto‐strain male rats, treatment for 4 weeks with defatted *T. molitor* meal showed no effects on food intake or BW.^19^ A longer treatment (90 days) in female rats, treated with 300–3000 mg kg^−1^ BW, also showed no effects on BW.^20^ Instead, increased food intake and BW were observed in obese Zucker rats fed a meal enriched in *T. molitor*, and this was remarkably accompanied by lipid‐lowering effects in the liver.^21^ Taken together, there is no clear consensus about the effects of *T. molitor* on food intake in rats. The varying results could be explained by different dosages and administration modes. Diverse results have also been found in farm animal species in relation to food intake, which either increased or decreased, although BW increase has often been reported.^22^, ^23^, ^24^, ^25^, ^26^ Further research to clarify the effects of *T. molitor* on food efficiency and the mechanisms behind it would be of interest in order to determine its potential use as an alternative protein source in situations of appetite loss.

To analyse the possible mechanisms for the food‐intake‐promoting properties, we studied the effect of insects on the enterohormone system. We previously found that secretion of GLP‐1 was stimulated by *A. diaperinus* in the human colon.^11^ Now, we have found that, in rat distal jejunum explants, treatment with *A. diaperinus* also stimulates GLP‐1 secretion. In the current study, we not only reproduced the GLP‐1 secretion in rat explants but also found an increase in plasmatic total GLP‐1 after administering the insects to the rats. This shows that the GLP‐1‐promoting effects also take place *in vivo*. Actually, *T. molitor* exerted the same GLP‐1 release, suggesting that this effect is not specific to the *A. diaperinus* species. Using the GLUTag cell line, we have shown that *A. diaperinus* acts directly on enterohormone cells to promote GLP‐1 release. Since the most abundant component of *A. diaperinus* is protein, we tested whether the GLP‐1‐releasing effects were due to the most abundant amino acids found in *A. diaperinus*. Our results showed that it was not. The effects then could be mediated by other amino acids, peptides, or fatty acids also found in the intestinally digested samples (for detailed *A. diaperinus* composition, see Accardo *et al*.^14^), which could be tested in the future. The lower amount of carbohydrates in this insect powder makes it very unlikely that the effects were due to glucose. In any case, GLP‐1 is a well‐described anorexigenic hormone, and its secretion does not explain the increase in food intake observed in *A. diaperinus*‐fed rats. In our experimental protocol we administered the protein load to food‐deprived animals at 6:00 p.m., and food was replaced 1 h after treatment. It has been previously shown that GLP‐1 does not induce satiety in fasted rats,^27^ so this would explain why the secretion of an anorexigenic hormone did not lead to a reduced food intake.

Our previous results showed that beef protein also stimulated GLP‐1 secretion in human explants; however, it differed from insect protein in that it did not modify colonic ghrelin secretion, whereas *A. diaperinus* significantly reduced it.^11^ Now we have shown a reduction in ghrelin secretion in rat distal jejunum after treatment with digested *A. diaperinus*. This result agrees with the fact that most metabolite G protein‐coupled receptors controlling ghrelin secretion are inhibitory, whereas all metabolite receptors controlling GLP‐1 secretion are stimulatory.^28^ This result is striking, since ghrelin is a well‐known orexigenic hormone. However, when there is a 60 min difference between ghrelin and GLP‐1 administration, it has been shown in mice that the prevailing action on vagal afferents and feeding response is that of the second hormone administered.^29^ Thus, the GLP‐1 secretion induced by food replacement might have overcome the effects of the lowered ghrelin levels 60 min earlier, annulling the effect of a reduced orexigenic signalling. Furthermore, it has been proposed that when ghrelin peaks before meals, as a food anticipatory hormonal response,^30^ this acts as a priming for glucose‐stimulated GLP‐1 secretion^31^ and sensitizes gut neurons to the actions of GLP‐1.^32^ According to this hypothesis, acute administration of insect protein before meals would reduce this ghrelin priming. Therefore, after food replacement, intestinal glucose‐stimulated GLP‐1 secretion and/or GLP‐1 sensing would be lower in insect‐treated rats than in control animals, and this reduced satiating signalling could lead to increased food intake, as we observed until the second week. We did not find a reduction in plasma ghrelin in the present study. This could be explained by a local effect of ghrelin priming in the small intestine that does not involve changes in peripheric ghrelin levels. However, another possible explanation could be the time points when it was analysed. Plasmatic levels at sacrifice were measured 22 h after the last insect dose, so the acute effects were not observed. Before the sacrifice, at week 13, we also measured ghrelin levels 20 min after the *A. diaperinus* dose. This is the time point at which the effect on GLP‐1 could be seen but when the effect on ghrelin was still not observed. Treatment with peptone, a meat hydrolysate that reduces ghrelin secretion in mice jejunum *ex vivo*, reduced plasma ghrelin levels 40 min after oral gavage.^33^ A similar profile was observed for ghrelin with other treatments 60 min after their administration.^34^ Actually, in another set of rats, we found that an acute treatment with *in vitro* digested *A. diaperinus* did not reduce ghrelin secretion in the duodenum, as it did in the distal jejunum of the control and *A. diaperinus*‐fed rats. This is not unusual, since amino acids and small peptides are sensed by the ghrelin cells that respond by activating or inhibiting ghrelin‐release pathways depending on the region in the gut.^33^ It has been shown that though the primary source of ghrelin is the gastric mucosa, small intestinal nutrient exposure is sufficient to decrease postprandial ghrelin levels.^35^ It has also been suggested that glucose, lipids, and a mixture of essential and non‐essential amino acids reduced ghrelin secretion through distal effects to the stomach and duodenum.^36^ Thus, it is possible that some components of *A. diaperinus* reached the jejunum *in vivo*, reducing intestinal ghrelin secretion, as observed *ex vivo*. Phenylalanine, which is abundant in *A. diaperinus*, has been reported to reduce active ghrelin *in vivo* in rats^37^ and *ex vivo* in mouse gastric mucosa.^38^

Although we did not find chronic effects on baseline or *A. diaperinus*‐stimulated enterohormone secretion, our results showed that the effects on stimulating food intake were lost after 1 week. We then hypothesized that the enterohormone sensing was altered after this week. Our results showed that there was no central effect on the GLP‐1 or ghrelin receptor gene expression. However, in the ileum, there was a tendency towards an increased messenger RNA of the ghrelin receptor, which makes sense with the hypothesis that the reduction in ghrelin might have local effects on the GLP‐1 priming system. Thus, there might be increased intestinal ghrelin sensing that compensates for the *A. diaperinus*‐lowered ghrelin levels, leading to a loss in effects on food intake.

## CONCLUSION

We showed for the first time the chronic effects of feeding rats with *A. diaperinus*. With our experimental administration protocol in a fasted state, 1 h before refeeding, *A. diaperinus* initially increased food intake. This could be explained by a local ghrelin reduction acting in the small intestine. These results indicate that insect protein could be useful for treating loss of appetite, although compensatory effects should be characterized further to avoid the loss of effects that we found in this study. Further experiments with humans should be performed to analyse whether the results vary compared with rats or if they are maintained.

## FUNDING INFORMATION

This research was funded by the Diputació de Tarragona (DIPTA 2020/26) and Proyecto AGL2017‐83477‐R funded by grant 248/10.13039/501100011033 MCIN/AEI/FEDER ‘Una manera de hacer Europa’. Alba Miguéns‐Gómez, Marta Sierra‐Cruz, and Helena Segú received a doctoral research grant from the Martí Franquès programme of University Rovira i Virgili. Montserrat Pinent and Ximena Terra are Serra Húnter fellows.

Nutritional composition of insect samples is available in Supporting Information Table S1.

## Supporting information


**Table S1.** Nutritional composition of the administered treatments measured on dry matter (values per 100 g insect flour).
Fig. S1. Diagram of the experimental design and the analysis performed. FI, food intake; BW, body weight; EE, enterohormone.
