# Supplementary material for: Administration of Alphitobius diaperinus or Tenebrio molitor before meals transiently increases food intake through enterohormone regulation in female rats
Source: J Sci Food Agric. 2022 Nov 21;103(4):1660–7. doi: 10.1002/jsfa.12305 (PMC10099498; doi:10.1002/jsfa.12305)
Supplement: Supplementary file 1 — Table S1. Nutritional composition of the administered treatments measured on dry matter (values per 100 g insect flour). Fig. S1. Diagram of the experimental design and the analysis performed. FI, food intake; BW, body weight; EE, enterohormone. [file JSFA-103-1660-s001.docx]

**SUPPLEMENTARY MATERIALS**

**Table S1.**Nutritional composition of the administered treatments measured on dry matter (values per 100 g insect flour).

| **Composition** | ***Raw***  ***A. diaperinus*** | ***Raw***  ***T. molitor*** |
| --- | --- | --- |
| Energy (kJ) | 2550 | 2604 |
| Protein (g) | 56.31 | 56.1 |
| Total lipids (g) | 18.82 | 26.31 |
| Starch (g) | 1.30 | 3.34 |
| Fibre (g) | 7.44 | 7.78 |


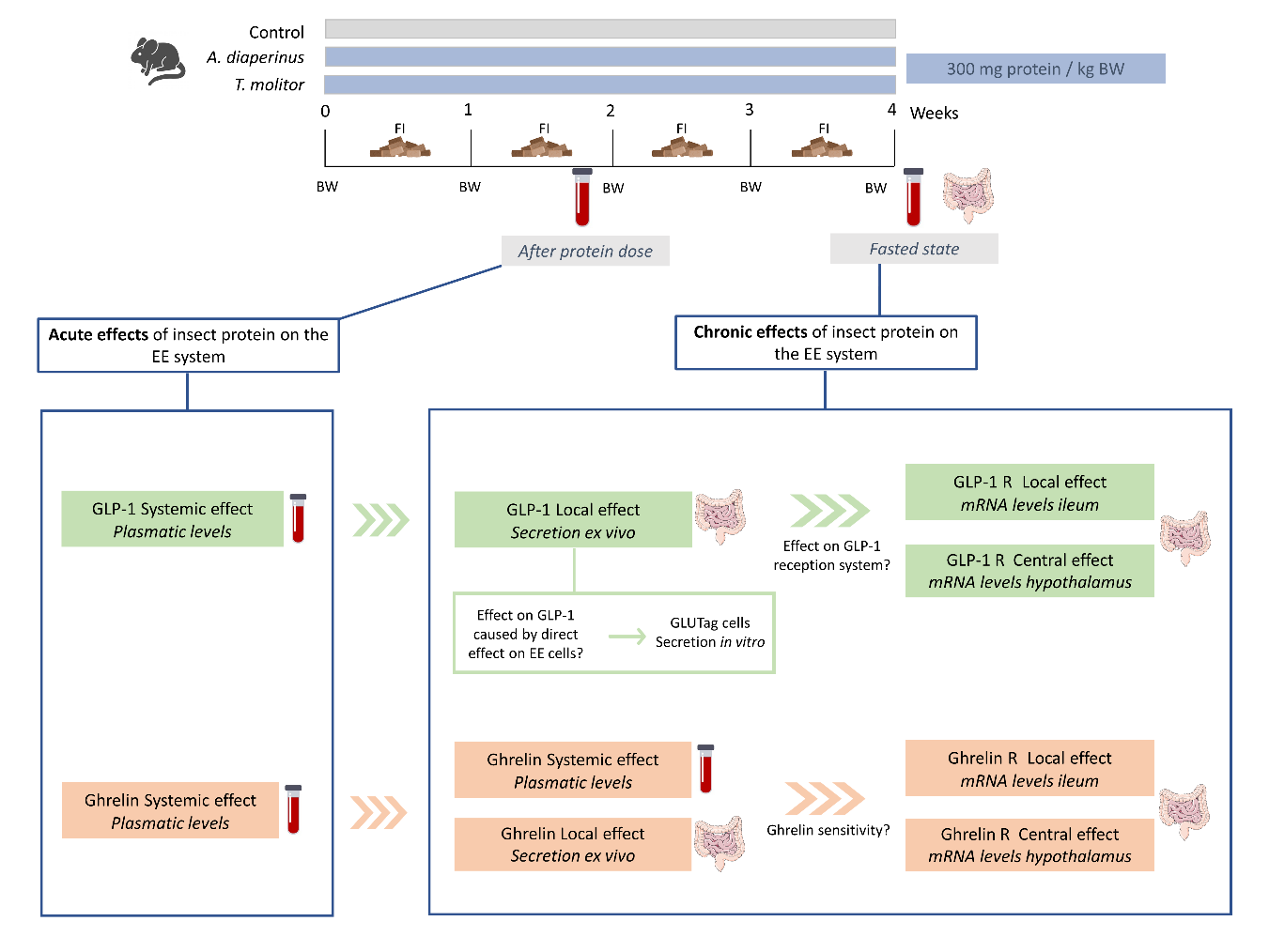


**Figure S1**. Diagram of the experimental design and the analysis performed. FI, food intake; BW, body weight; EE, enterohormone.
